# Some lessons from COVID: science and communication

**DOI:** 10.1107/S2052252521003894

**Published:** 2021-04-15

**Authors:** Edward N. Baker

**Affiliations:** a School of Biological Sciences, University of Auckland, Auckland, New Zealand

**Keywords:** COVID-19, coronaviruses, SARS-CoV-2, scientific communication, editorial

## Abstract

Biomedical challenges such as the present COVID-19 pandemic require both good science and excellent communication between scientists and the general public. This underscores the importance of presenting our science in innovative ways that make it accessible to all.

What a turbulent and (for many) traumatic year we have experienced since my editorial of last May (Baker, 2020 ▸)! Responses around the world have varied greatly as nations sought to find a way through the current COVID-19 pandemic, and there are surely many lessons to be learned by all of us. My vantage point, as I write, is a rather privileged one, living as I do in a country in which there have been very few deaths (26 in a population of 5 million). We were fortunate in having time to observe the spread of the pandemic in other countries before it could take hold seriously here. But some aspects of our response are of universal relevance.

A key factor in New Zealand’s experience was the excellence of scientific communication, and the willingness of both the government and the general public to listen. Scientists had direct access to government and a strong role in policy development, and daily briefings by the Prime Minister and our Director-General of Health became essential listening. Some superb science communicators emerged, one of whom, Dr Siouxsie Wiles, a microbiologist from the University of Auckland, has recently been chosen as New Zealander of the Year. It seems to me that one outcome – here at least – has been a thirst for scientific information, and an accompanying rise in the estimation of science. Many more people now know what a virus looks like, and can talk about the ‘spike protein’. And also know that proteins are much more than something you eat!

How do these issues impact on scientific publication, and on open-access journals such as **IUCrJ** in particular? It is in the speed with which an open-access article addressing a topic of major public interest can become widely disseminated. Almost immediately after publication of my 2020 editorial on the rapidity of the structural biology response to the COVID pandemic, I found substantial portions being quoted on Facebook and began receiving emails from family and friends from all around the world. Likewise, Wladek Minor, lead author on an article in the present issue of **IUCrJ** (Grabowski *et al.*, 2021 ▸), tells me that he has never before received such immediate worldwide attention and interest. His article focused on protocols and tools to enable more effective responses to future biomedical threats.

At the same time, this mobilization of structural biology to address the COVID pandemic poses real challenges of communication. There has been explosive growth in the number of structures solved for components of the SARS-CoV-2 virus. At time of writing, the worldwide Protein Data Bank (wwPDB) contains more than 1000 such structures, all solved in the past year. These include 345 structures of the spike protein (the principal target of vaccines), exploring conformational variations, effects of mutations, and the binding of ligands; 300 structures of the main protease, a key drug target, with and without ligands or drug candidates bound; and 489 other structures of proteins, RNA *etc*. So far, few of these have been published in journal articles – the days of one structure, one paper, are long gone! Often only the first few structures for a given protein are published, *e.g.* for the SARS-CoV-2 spike protein (Walls *et al.*, 2020 ▸; Wrapp *et al.*, 2020 ▸) or for the main protease 3CL^pro^ (Zhang *et al.*, 2020 ▸; Jin *et al.*, 2020 ▸).

A rich resource remains, however, and the challenge is to sort through these data to extract new and interesting insights. Several such papers have been published in **IUCrJ** over the past year. One such, a retrospective cryo-EM analysis, reveals the considerable flexibility that exists in the spike protein in its pre-fusion state, before engagement with its cell receptor (Melero *et al.*, 2020 ▸). Another (Jaskolski *et al.*, 2021 ▸) compares and analyses 81 crystal structures of the main protease 3CL^pro^ from SARS-CoV-2 to assess their quality and consistency, with a view to establishing firm bases for drug development. I feel sure that many more issues of significant public interest can be brought forward from these structural data, for example on the structural effects of mutations and how these may influence vaccine efficacy or drug development.

As a final comment I encourage our writers to bring their science forward in ways that are more accessible to the general public. We could certainly expand the number of Scientific Commentaries we publish, but I am thinking of something briefer and more accessible to all, in the form of short news items, informed by science. There is certainly an appetite for that at the present time.
